# Centrifugal Compressor Stall Control by the Application of Engineered Surface Roughness on Diffuser Shroud Using Numerical Simulations

**DOI:** 10.3390/ma14082033

**Published:** 2021-04-18

**Authors:** Amjid Khan, Muhammad Irfan, Usama Muhammad Niazi, Imran Shah, Stanislaw Legutko, Saifur Rahman, Abdullah Saeed Alwadie, Mohammed Jalalah, Adam Glowacz, Mohammad Kamal Asif Khan

**Affiliations:** 1 Faculty of Mechanical Engineering, National University of Technology, Islamabad 44,000, Pakistan; imranshahswabi@gmail.com; 2 Electrical Engineering Department, College of Engineering, Najran University Saudi Arabia, Najran 61441, Saudi Arabia; miditta@nu.edu.sa (M.I.); srrahman@nu.edu.sa (S.R.); asalwadie@nu.edu.sa (A.S.A.); msjalalah@nu.edu.sa (M.J.); 3 Faculty of Mechanical Engineering, Poznan University of Technology, 3 Piotrowo Str., 60-965 Poznan, Poland; 4 Faculty of Electrical Engineering, Department of Automatic Control and Robotics, Automatics, Computer Science and Biomedical Engineering, AGH University of Science and Technology, al. A. Mickiewicza 30, 30-059 Kraków, Poland; adglow@agh.edu.pl; 5 Mechanical Engineering Department, College of Engineering, Najran University Saudi Arabia, Najran 61441, Saudi Arabia; mkkhan@nu.edu.sa

**Keywords:** centrifugal compressor, numerical simulations, surface roughness, stall margin, flow instability

## Abstract

Downsizing in engine size is pushing the automotive industry to operate compressors at low mass flow rate. However, the operation of turbocharger centrifugal compressor at low mass flow rate leads to fluid flow instabilities such as stall. To reduce flow instability, surface roughness is employed as a passive flow control method. This paper evaluates the effect of surface roughness on a turbocharger centrifugal compressor performance. A realistic validation of SRV2-O compressor stage designed and developed by German Aerospace Center (DLR) is achieved from comparison with the experimental data. In the first part, numerical simulations have been performed from stall to choke to study the overall performance variation at design conditions: 2.55 kg/s mass flow rate and rotational speed of 50,000 rpm. In second part, surface roughness of magnitude range 0–200 μm has been applied on the diffuser shroud to control flow instability. It was found that completely rough regime showed effective quantitative results in controlling stall phenomena, which results in increases of operating range from 16% to 18% and stall margin from 5.62% to 7.98%. Surface roughness as a passive flow control method to reduce flow instability in the diffuser section is the novelty of this research. Keeping in view the effects of surface roughness, it will help the turbocharger manufacturers to reduce the flow instabilities in the compressor with ease and improve the overall performance.

## 1. Introduction

Automotive turbochargers consist of two parts; turbine and compressor. Centrifugal compressor is the fundamental part, which is an imperative component of the automation and production industry [1]. Centrifugal compressors have certain advantages over the axial compressors, such as light weight, high pressure ratio and compact structure. Due to these advantages, the demand is rapidly increasing in automotive industry, which challenges the manufacturers to not only improve the pressure ratio and efficiency but also amplify the operating range and ensure stable operation over the entire speed-line [2,3]. Downsizing in engine size is pushing the automotive industry to operate compressors at low mass flowrate [4]. However, at low mass flow rate the centrifugal compressor leads to fluid flow instabilities, which can increase aerodynamic loss and structural failure of impeller blades.

Commonly, there are two types of flow instabilities, stall and surge. Stall is flow separation phenomena that occurs at low mass flow rate due to flow separation and flow recirculation and it occurs before surge [3]. Surge is an aerodynamic flow instability, which occurs due to varying wild pressure fluctuation at low mass flow rate due to oscillating fluid flow with varying frequency. A flow near the wall may separate because of adverse pressure gradient, which leads to flow recirculation. As this flow leaves the wall, reverse flow starts to occur near the surface of the wall due to adverse pressure gradient. When stall divides into many stall cells, it propagates in circumferential direction with a lesser speed than the rotational speed of the impeller [4]. There are two main types of stall initiations during transient simulations: modal-type and spike-type [5,6]. At the peak of the stagnation to static pressure rise, the modal type of stall appears to occur while spike type stall inception usually diffuses much faster than modal type stall. Spike type stall occurs as a crest in the pressure curve and trough spike in the velocity curve during unsteady simulations [6,7].

Greitzer et al. [8] experimentally investigated surge phenomena and concluded that it can be divided into mild and deep surge based on the back-flow condition. Mild surge usually causes small flow oscillation and it decreases efficiency with no back flow, however, deep surge leads to catastrophic failure of the system with drastic oscillation in mass flow, which creates extremely high noise and back flow [9].

Past research on the theories of flow instabilities that arise in the centrifugal compressor, show the instability boundaries. Greitzer et al. [8] developed a one-dimensional model which was used to show the flow instability in the compressor, which will either enter the state of stall or surge. Moores et al. [6] developed a theory, which indicated that rotating stall or surge is because of a wave having higher order propagation in the form of peaks. Zheng and Liu [10] developed the mass spring damping system to detect stall and surge structures at different locations. It was concluded that the flow leakage from the tip clearance leads to backflow and stall in the compressor.

Experimental and numerical investigations have been performed to control the compressor instabilities. To reduce flow separation in the impeller, the application of surface roughness on the impeller-blades and impeller-inlet is one of the efficient methods to control stall. These impeller components contribute maximum to the entropy generation but convey the most sensitivity to deviation in surface roughness [11]. As the mass flow rate decreases near stall point, the high angle of incidence at the impeller outlet produces flow recirculation and flow separation that leads to ordinary compressor stall or surge [12]. To reduce the flow separation at impeller exit, Xiang et al. [2] experimentally investigated the influence of variable vanes installed on a diffuser shroud. It was concluded that when the air flow angle of incidence increases at the inlet of diffuser, the aerodynamics load decreases and the flow structure of the diffuser vaneless region improves significantly. Yingji et al. [13] studied the parametric analysis of slotted diffuser geometry variation to augment the stable operating range of the high-pressure ratio centrifugal compressor. Their analysis concluded that at reduced mass flow rate, there is flow separation at the corner of diffuser hub, which has been eliminated by the length of slot, which was 8–16% of chord length of diffuser without significant deterioration in performance.

Flow separation leads to extremely high vibration and high pitch noise, causing the compressor to catastrophic failure due to fatigue load. To control flow separation, turbulent boundary layer is important to study. Turbulent boundary layer is the subject of the experimental and numerical studies due to its eminence in the automotive, aerospace, and industrial flows applications [14]. The relative velocity for no slip conditions is zero and for a rough wall, the protruded roughness height makes the fluid flow field extremely complex. In each case of surface roughness, the rough surface reveals itself as typical vortical pattern, which is universal for turbulent flow near the wall [15].

Being prominent as a passive flow control method in many engineering applications, rough surface turbulent flows have extensively been studied by Promode R. et al. [16], G. I. Barenblatt [14] and Krogstad et al. [17] and Raupach et al. [18]. One of most important factors to study during surface roughness analysis is wall shear stress, or the frictional velocity.

Barrenblatt et al. [14] has presented the theoretical opinions supporting the power law velocity profile in the overlap region. However, logarithmic velocity profile has been traditionally used for various mechanical applications for a longer period of time. In fact, the rough surface makes the flow pattern too complex. When roughness element is applied, the flow is no longer parallel to the wall on micro scale [14]. In general, the flow in the roughness sublayer is non homogenous as compared to the viscous sub-region of smooth wall. Isaacson et al. [19] studied that for high Reynolds number, as is the case of baseline compressor, the turbulent flow outside the roughness sublayer is not affected by the roughness. A very simple and practical way to study fully rough boundary layer is to substitute the viscous length scale of smooth wall with the constant mean roughness height, K.

The aforementioned studies have been able to highlight the superior methods to control stall phenomena; however, those flow control methods were extremely complex for manufacturers to shape in mass production due to high manufacturing cost and unavailability of production unit. Moreover, to study the flow pattern responsible for the compressor stage performance fluctuation is also relatively insufficient. By considering the limitation of past studies, this research has been performed numerically to improve the performance, flow structure, to control compressor instabilities and facilitate manufacturers with simple passive flow control method. In this research paper, surface roughness as a passive flow control method is applied on a diffuser shroud used to control stall using numerical simulations and its effect on the performance, operating range and flow structure has been studied.

The objective of this research is to numerically examine the effect of surface roughness on the boundary layer, flow structure and the flow instability near diffuser shroud of centrifugal compressor. A systematic approach has been followed for this analysis, which includes the application of surface roughness on diffuser shroud by: (i) application of surface roughness on single passage of diffuser shroud, and (ii) then integrate the performance for the whole compressor. The main objectives are: (a) perform a 3D steady CFD analysis to study the effects of stall for SRV2-O compressor and surface roughness effect on flow instability, (b) to increase the operating range of centrifugal compressor, (c) study boundary layer flow for smooth and rough surface, and (d) study the effect of surface roughness on performance parameters such as pressure ratio, entropy generation and isentropic efficiency.

### Scope of the Paper

**Figure 1:**
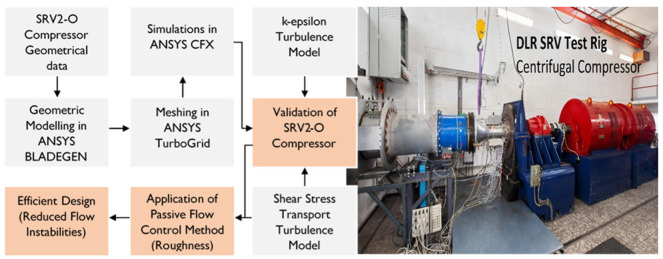
Methodology developed for the analysis of SRV2-O turbocharger compressor (**left**) and DLR SRV compressor test rig (**right**) (Image courtesy: DLR (CC-BY 3.0) (German Aerospace Center)) Adapted with permission from ref. [31].

Figure 1 depicts the methodology used to illustrate the influence of surface roughness on the performance and operating range of the centrifugal compressor. The baseline compressor specifications are taken from the DLR SRV2-O compressor including number of blades, diameter of the impeller and diffuser, inlet and outlet angles of flow, and inlet boundary conditions are listed in Table 1 [20]. The theoretical background of surface roughness, types of different roughness regions, and its effect on flow structure has been elaborated in Section 2. Section 3 describes the numerical setup, which includes geometric parametrization in BladeGen, meshing in TurboGrid, boundary conditions and turbulence model. In Section 4 of the paper, available experimental results for the baseline compressor SRV2-O are validated using numerical simulations with the detailed topography of the flow and discusses the influence of different surface roughness magnitudes on the operating range, maximum entropy generation regions, and flow instabilities in the compressor. A comparison is made between the available experimental data from DLR and the simulated baseline configuration. The conclusion that emerged from this research is described in the Section 5 to provide a summarized form for the better understanding of the influence of surface roughness on compressor performance.

## 2. Baseline Compressor Specifications

A baseline centrifugal compressor SRV2-O having high specific speed, flow rate and pressure ratio has been simulated to study the parametric analysis of surface roughness of different magnitudes on flow behavior and performance. The baseline compressor for the current study was developed and designed by German Aerospace Center, Cologne, Germany (Deutsches Zentrum für Luft- and Raumfahrt; DLR). The technical specifications are listed in Table 1.

The baseline impeller consists of total of 26 blades with 13 full and splitter blades. At the impeller outlet domain, a vaneless diffuser is attached, and this diffuser completes the compressor stage with overall radius of 179 mm. The compressor for the current analysis has outer radius of 112 mm with a splitter blade leading edge starts at 55 mm radial direction and impeller rotational speed of 50,000 rpm. The design tip clearance is variable tip clearance, 0.05 cm at the blade leading edge and 0.03 cm at the blade trailing edge. The impeller (SRV2-O) design data are shown in Table 1 [21]:

### The Wall Surface Roughness

Table 2 shows the roughness magnitudes specified to be applied on the diffuser shroud. Based on the previous literature, the smooth metal surface has normally roughness of about 3.18 μm [22]. In high Reynolds number applications, surface roughness effect is used to increase the speed of transition from laminar-turbulent and delay flow separation [23]. Therefore, in order to reduce flow reversal and flow recirculation, roughness of height 50 to 200 μm has been applied. In order to assign geometric surface roughness magnitude to combine with equivalent roughness, Simon et al. [24] formulated the following correlation:

K_s_ = 2R_a_ (1)

where R_a_ stands for the centerline average roughness value and k stands for geometric roughness magnitude [25]. Surface roughness has not only an effect on the flow structure and pattern but also influences the operating properties of the machine parts [26].

Local flow separations in the flow structure occur at the rough surfaces. During flow separation, wakes are created on the surface of diffuser shroud and this vortex is counter rotating vortex generating pairs. Roughness element’s size is presented based on length scale of the vortices that causes separation of flow from the wall of the compressor shroud. Now, the sublayer induced by surface roughness near the wall of the compressor mixes into the flow. The low Reynolds number flow suppress the vortices/wakes with the help of viscosity but at high Reynolds number these wakes speed up the transition process from laminar to turbulent. Surface roughness has an effect on both the compressors and turbines flow structure. Based on the general trends it is still beyond our ability to predict the roughness effects on losses due to fluid friction and surface heat transfer accurately [27]. There are still very large gaps in research to properly characterize roughness e.g., size, shape, spacing and roughness location. Equivalent sand grain roughness characterization (ks) has impeded comprehensive use for modelling since it does not account for different roughness effects on heat transfer, skin friction and boundary layer transition [23].

Prakhar et al. [28] closely investigated the effect of roughness on different surfaces on turbulent flow structure. The dependencies of different flow regions on Reynolds number and different surface roughness were obtained from the Nikurasde pressure loss data. In his study on surface roughness, based on the diameter of sand grain (Ks), dynamic viscosity (υ) and wall friction velocity (V*), dimensionless parameter K+ = (Ks V*)/υ was defined. The loss coefficient is only a function of ks for a value of this parameter greater than 70. The parameters Re and K+ becomes too much important when the value of ks is between 5 and 70 [28]. Nikurasde defined three different regions based on his analysis:

- Hydraulically smooth region (0 < K+ < 5), in this region the size of the grain is too small and it has no significant impact on the performance of the compressor;
- Transition region (5 < K+ < 70), in this region the sand grain/roughness elements are great enough to project out of closest layer to wall and further resistance is observed because of high surface roughness;
- Completely rough region (K+ > 70), in this region all the sand grain/roughness heights project out of the viscous sublayer and drag form of resistance is offered.

Schlichting et al. further elaborated and formulated the concept of sand grain roughness [29]. Schlichting et al. used the concept of equivalent sand grain roughness for converting the roughness data and measurement equivalent to the roughness data used by prakhar et al. [28] corresponding to several more flow profiles into roughness profiles.

In our analysis, all three different regions of surface roughness developed by Nikurasde have been investigated to study the effect of each roughness region on the flow structure and flow instability in the diffuser. Magnitude of applied roughness has been mentioned in the Table 2.

## 3. Numerical Setup

For CFD model to acquire, numerical simulations have been performed following step by step methodology shown in Figure 1. For numerical analysis, ANSYS version 15.0 is used [30]. The stepwise numerical setup was followed, such as modelling of compressor in BladeGen, creating mesh in TurboGrid, defining the turbulence models, and feeding the boundary conditions to ANSYS CFX.

### 3.1. Modelling in BladeGen Using Geometric Parametrization

**Figure 2:**
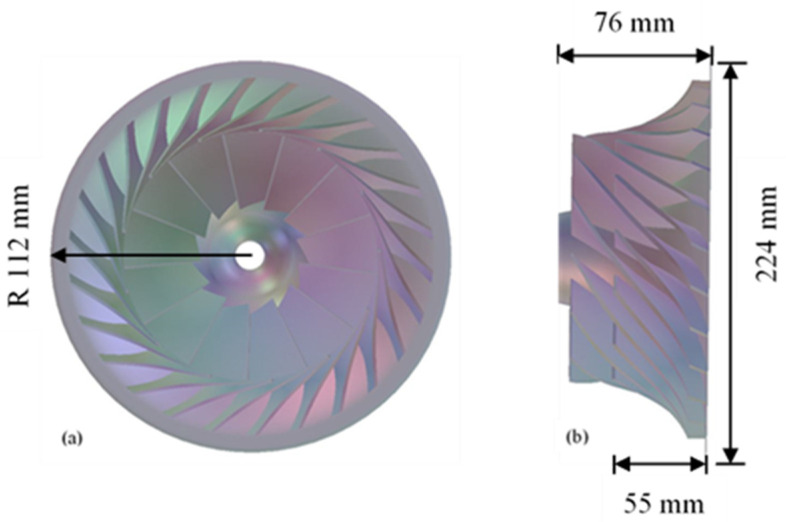
Bladegen model of the impeller (**a**) Front view (**b**) Side view.

The initial step is modelling accurate compressor in the BladeGen module of the ANSYS using the data provided by the German Aerospace center to acquire proper design as shown in Figure 2a,b [32]. BladeGen tool is used for radial geometries, which examines 2D data and convert it into 3D model, which provides the most accurate radial geometries for simulation purposes. In BladeGen module, it assembles all the components to represent it as a single domain [31].

### 3.2. Meshing in TurboGrid

**Figure 3:**
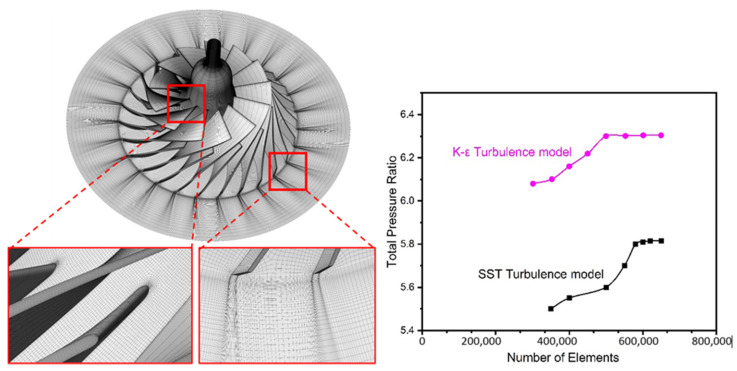
Computational grid (**left**) and mesh independency test (**right**).

Compressor is 3D modelled in the BladeGen module of the compressor, then an H-grid mesh type has been created for the reference and baseline SRV2-O compressor in the TurboGrid module of ANSYS. TurboGrid is specifically used for the turbomachinery setups, this module creates refined hexahedral mesh automatically for all kinds of complex turbomachinery applications. As validation is carried out using both k-ε and shear stress transport turbulence models, mesh properties have been defined accordingly. To study k-ε turbulence model, y-plus value is taken as 35 while for shear stress transport model y-plus value of 0.001 is defined for this turbulence model. H-grd topology is used for both the turbulence models with 30 elements in each inlet and outlet. A grid independency test showed that the adequate number of mesh elements for the k-epsilon model and shear stress transport model are 560,788 and 602,613, respectively, as there was no significant change in the pressure ratio of the compressor as illustrated in Figure 3 [33].

### 3.3. Turbulence Model

To design a flow field for the steady state numerical simulations, ANSYS CFX 15 is employed. In the ANSYS CFX module, two turbulent flow models to capture turbulence in the flow which are k-epsilon (k–ε) and SST (Shear Stress Transport) models are mostly used turbulence models. The two equations k-epsilon model is normally used to capture turbulence in engineering flow problems. However, this turbulence model cannot be used when it comes to modeling complex fluid flow problems involving high adverse pressure gradients and separation. The primary reason is that it is not able to predict the complex fluid flow pattern with sufficient accuracy. On the other hand, to overcome the limitations of k-epsilon, the SST model is used to capture the boundary layer formation and subsequent separation.

In the current study, both SST and k-ε models are used for the numerical investigation of the baseline compressor. The computational cost per iteration increases as the number of equations and parameters in the models increases, so does the accuracy [34].

### 3.4. Boundary Conditions and Numerical Setup

**Figure 4:**
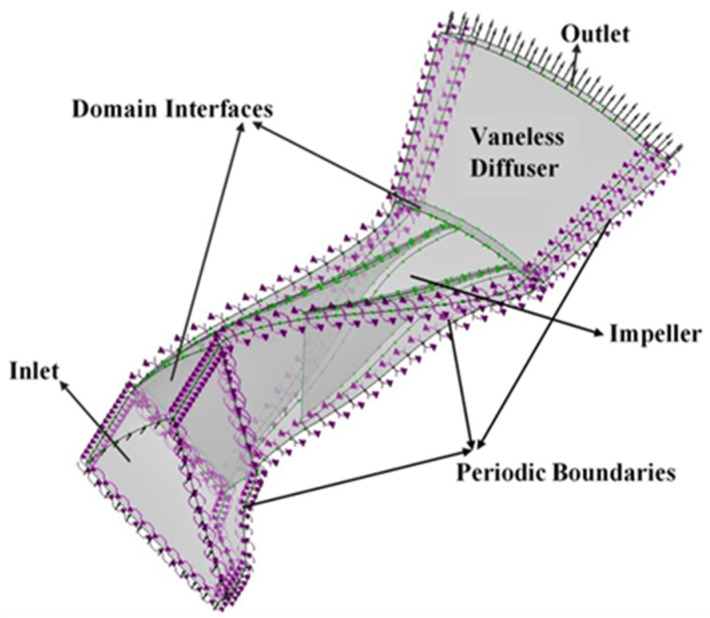
Computational domain.

The boundary conditions applied to the baseline SRV2-O compressor is kept the same as experimental conditions. The boundary conditions specified at inlet of compressor are: T_i_ = 288.15 k, P_i_ = 101.325 kpa and convergence criteria is 10^−6^. According to standard ambient conditions, the medium turbulence intensity is kept at 5%, which was used by the experimental setup at DLR for turbo setup. The SIMPLE pressure velocity algorithm has been applied with a high-resolution advection scheme and first order upwind discretization to all solution variables. Mass flow rate is considered for the convergence, which is recorded by slowly decreasing static pressure at outlet and the convergence criterion is given a value of 1 × 10^−6^ for all residuals. As it is a well-known fact that pressure ratio is the smallest at choke point, so static pressure is specified in the expressions section of the CFX preprocessing, which is used to find the choke point for the compressor speed line. For interfaces between inlet, impeller and diffuser, stage interfaces for steady state analysis have been defined. All the walls are considered as hydraulically smooth walls and surface roughness has been defined on diffuser shroud for later analysis. The rotational periodicity has been defined across all the passages as shown in Figure 4 [35].

## 4. Results and Discussion

### 4.1. Validation of Experimental Data

To assess the performance of centrifugal compressor, the parameters that are analyzed are the pressure ratio and isentropic efficiency against mass flow rate. This analysis is carried out from compressor inlet to diffuser comparing the baseline SRV2-O compressor between the experimental results and CFD predicted results at design speed of 50,000 rpm.

**Figure 5:**
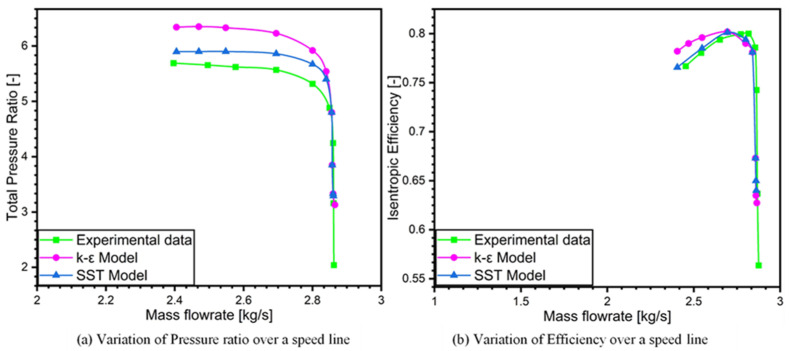
Performance map for pressure ratio and efficiency (**a**) Variation of pressure ratio over a speed line (**b**) Variation of efficiency over a speed line.

To numerically simulate the 3D model, several agreements are made for CFD solvers with the experimental calculations, which are listed in bullets [36]:

- K-ε and SST Turbulence models are used for validation;
- Simulations conditions: mass flowrate 2.55 kg/s and design rotational speed;

Numerical simulations are performed for the speed line from stall to choke at design rotational speed to compare with the experimental data. This comparative analysis is carried out to show the suitability of this solver (ANSYS CFX) and evaluate realistic results for further investigation and research in the baseline SRV2-O compressor. This comparison has been made based on simulated speed line for the baseline compressor with experimental speed line by assessing pressure ratio (outlet pressure/inlet pressure) and efficiency of the compressor. The comparison showed reasonable results, indicating that this method is able to predict the flow structure of the high-pressure ratio centrifugal compressor. The simulated and experimental speed line is illustrated in Figure 5. The numerical computations biggest discrepancy was the overprediction of the experimental results by 8% using k-epsilon model while 2.3% using k-omega SST turbulence model.

As it is steady state analysis, therefore, stall occurs either because of singular or three-dimensional separation. Singular separation occurs at low mass flow rate due to adverse pressure gradient, due to which recirculation of flow happens. The primary reason for three-dimensional flow separation is due to the existence of secondary flow. Secondary flow occurs because of pressure gradient in cross flow channel direction. At specific rotational speed, the mass flow rate decreases near stall and blade inlet flow angle rises. This results in increasing the incidence angle. This increase in incidence flow angle accelerate flow at the blade leading edge causing strong diffusion, which results in flow separation from the surface. On the other hand, choke occurs when compressor operates at maximum possible mass flow rate.

Figure 5 illustrates the efficiency performance speed line considering mass flow rate and total pressure ratio using k-ε and SST turbulence models. It is evident that the results are comparable to experimental data trends. Although CFD predicted the stall at high pressure ratio than that of experimental data of SRV2-O, however, the compressor map showed the same trend as the experimental data.

It could be concluded from the present investigation that the numerical simulations are proficient at showing the performance variation, flow and loss mechanism inside the roughened diffuser in high pressure ratio centrifugal compressor. This aberration from experimental data can be imputed to the shortcomings of the turbulence models and numerical errors. Thus, numerical computations can be used to study the performance variation with the application of surface roughness. The operating range from stall to choke for both computational and experimental result is 16%.

SST k–ω is used where highly accurate resolution of boundary layer is critical, i.e., applications involving flow separation of finely resolved heat transfer profiles. It is also found from the validation results that SST turbulence model shows best agreement with the overall experimental results as it overpredicts the experimental results by only 2.3%. Therefore, in the current study k–ω Shear Stress Transport (SST) is used for the rest of surface roughness analysis, which is able to capture flow near the wall surface as well as in the far field with great accuracy.

### 4.2. Influence of Surface Roughness on the Diffuser Flow Structure

**Figure 6:**
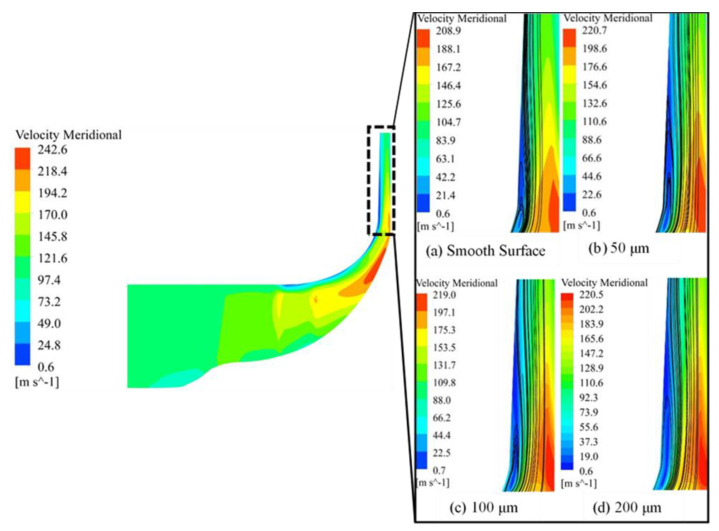
Diffuser meridional plane (**a**) Smooth surface (**b**) 50 μm surface roughness (**c**) 100 μm surface roughness (**d**) 200 μm surface roughness.

By computing the centrifugal compressor performance with three different types of surface roughness magnitudes, the impact of surface roughness on the flow instability and performance is summarized in the Figure 6. The most prominent features of Figure 6 are that diffuser roughness magnitudes result in slight increase in frictional losses relative to baseline SRV2-O compressor. The reason is that these roughness magnitudes slightly project out of the wall surface, increasing the drag resistance to the flow as addressed in the Section 2. Furthermore, the figure also illustrates the comparison of smooth surface with engineered rough surface. Evident from Figure 6, flow separation is significant in the case of smooth shroud surface, while it eliminates the flow recirculation effect when roughness magnitude increases from 50 μm to 200 μm. This flow instability at diffuser inlet is because of unsteady and viscous flow. At the impeller exit, strong fluctuations in the flow angles and the meridional velocity creates extremely complicated flow pattern at diffuser inlet as shown in Figure 6. It could be seen in Figure 6 that at the blade pressure side, jet flow structure exists which results in the flow-reversal accumulated near the shroud surface of diffuser. This jet flow region on the blade pressure side is considered as loss free but it creates low velocity regions at impeller outlet or diffuser inlet, which transports turbulence, frictional losses and accumulates near the shroud of the diffuser. This gives better explanation of the flow-reversal and flow-separation on the diffuser shroud surface.

Figure 6a further illustrates that shroud surface of compressor when studied smooth wall, a back-flow section was found on the shroud of the diffuser. The application of engineered roughness magnitudes has substantial effect on the performance and flow structure of the compressor. As roughness magnitude has been increased from smooth surface to 200 μm, a considerable decrease has been found in the flow reversal and flow separation at the impeller outlet/diffuser shroud and hence, suppressed stall phenomena as seen in Figure 6d. From the current analysis, significant reduction in flow separation is observed in the roughness case of 100 μm and 200 μm. The augment in surface roughness on the diffuser shroud causes the reattachment of flow and therefore reduces flow reversal on the diffuser shroud surface. Figure 6b–d shows the influence of the increasing roughness heights on the diffuser shroud in order to reduce flow-reversal of the compressor flow field. The increase in the surface roughness eliminates the flow reversal and flow separation of the reversed flow region of diffuser shroud as shown in Figure 6d.

Figure 6a is the performance map of pressure ratio against mass flow rate and Figure 6b shows the performance map of isentropic efficiency against the mass flow rate for a compressor stage of turbocharger. It is evident from Figure 6 that application of surface roughness has a great impact on the compressor performance. A steady state simulation has been executed at stall mass flow rate of 2.366 kg/s and at rotational speed of 50,000 rpm to evaluate the performance for centrifugal compressor at smooth diffuser shroud. The size of the roughness elements is increased at the same design condition from smooth surface to roughness magnitudes of (50 μm, 100 μm, 200 μm) and compared with the baseline compressor, which indicates that stall margin has been increased in each case of roughness heights by simulating it using SST turbulence model. However, there is drastic drop in total pressure ratio and isentropic efficiency with the increase in the roughness heights as compared to the slotted diffuser studied by Yingjie et al. [13]. The most significant drop in pressure ratio was observed when roughness magnitude rose to 200 μm.

**Figure 7:**
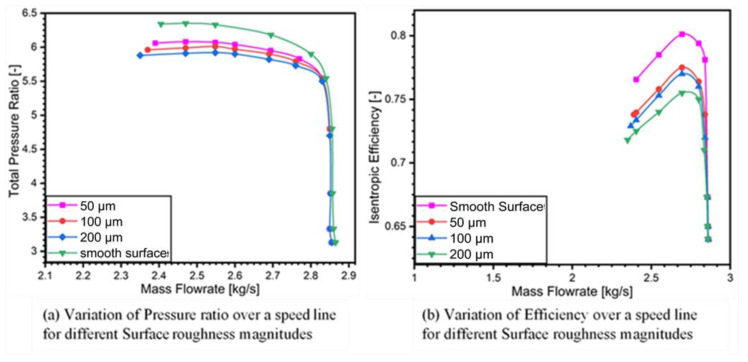
Performance maps for different roughness magnitudes (**a**) Variation of pressure ratio over a speed line (**b**) Variation of efficiency over a speed line.

The drop in pressure ratio has started when the roughness magnitude has been incrementally increased from 50 μm to 200 μm as shown in Figure 7a. This loss in pressure ratio is attributed to the frictional losses and thick boundary layer in the diffuser section, which leads to increase in surface blockage.

**Figure 8:**
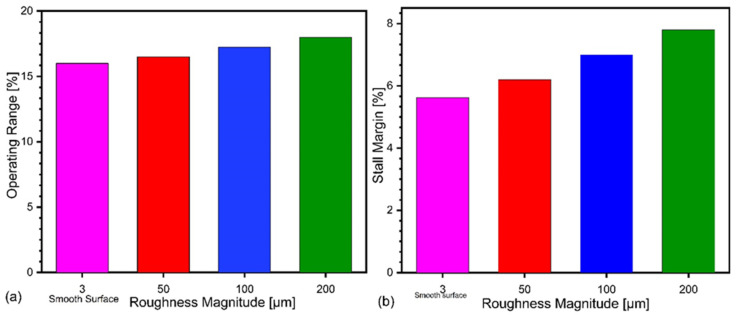
Influence of roughness magnitude on operating range (**a**) stall margin (**b**).

**Figure 9:**
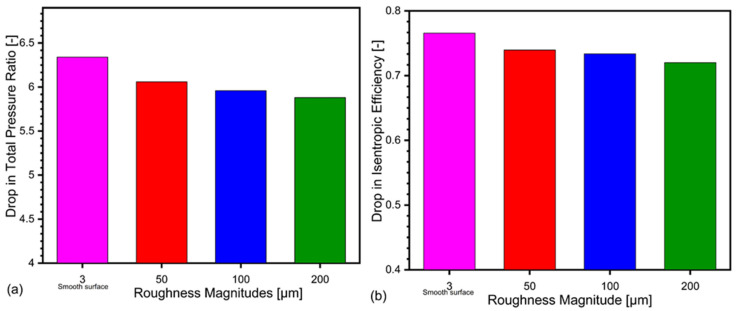
Drop in pressure ratio (**a**) and isentropic efficiency (**b**) with rise in roughness magnitude.

To further estimate the effect of surface roughness on the performance of centrifugal compressor, Figure 8 and Figure 9 depicts the statistical comparison of the examined surface roughness implemented diffusers with respect to stall margin, operating range, total pressure ratio and isentropic efficiency.

As illustrated in Figure 8, compared to that of baseline SRV2-O compressor, the stall margin is increased by the application of surface roughness on diffuser in each case of increased roughness magnitude results in increase of stall margin. This improvement in stall margin is at the loss of pressure ratio and efficiency. Stall margin for the compressor increased from 5.67% to 7.88% as shown in Figure 8a. The operating range for smooth case is 16.06%, and it increased to 18% for the roughness magnitude of 200 μm as shown in Figure 8b.

The influence of surface roughness on the performance of centrifugal compressor can be evaluated by the relative total pressure ratio and change in isentropic efficiency at the stall mass flow rate (m = 2.405 kg/s). Figure 8 shows the variation of total pressure ratio and isentropic efficiency related to variation of surface roughness magnitudes. The bars in the figure show that as the roughness magnitude increases, the pressure ratio and isentropic efficiency decreases steadily. The percentage drop in pressure ratio at stall point for 50 μm is 3.3%, 4.2% for 100 μm and 5.08% for 200 μm using k-ω SST turbulence model as shown in Figure 9. However, further increase in roughness magnitude deteriorate the compressor performance more significantly.

**Figure 10:**
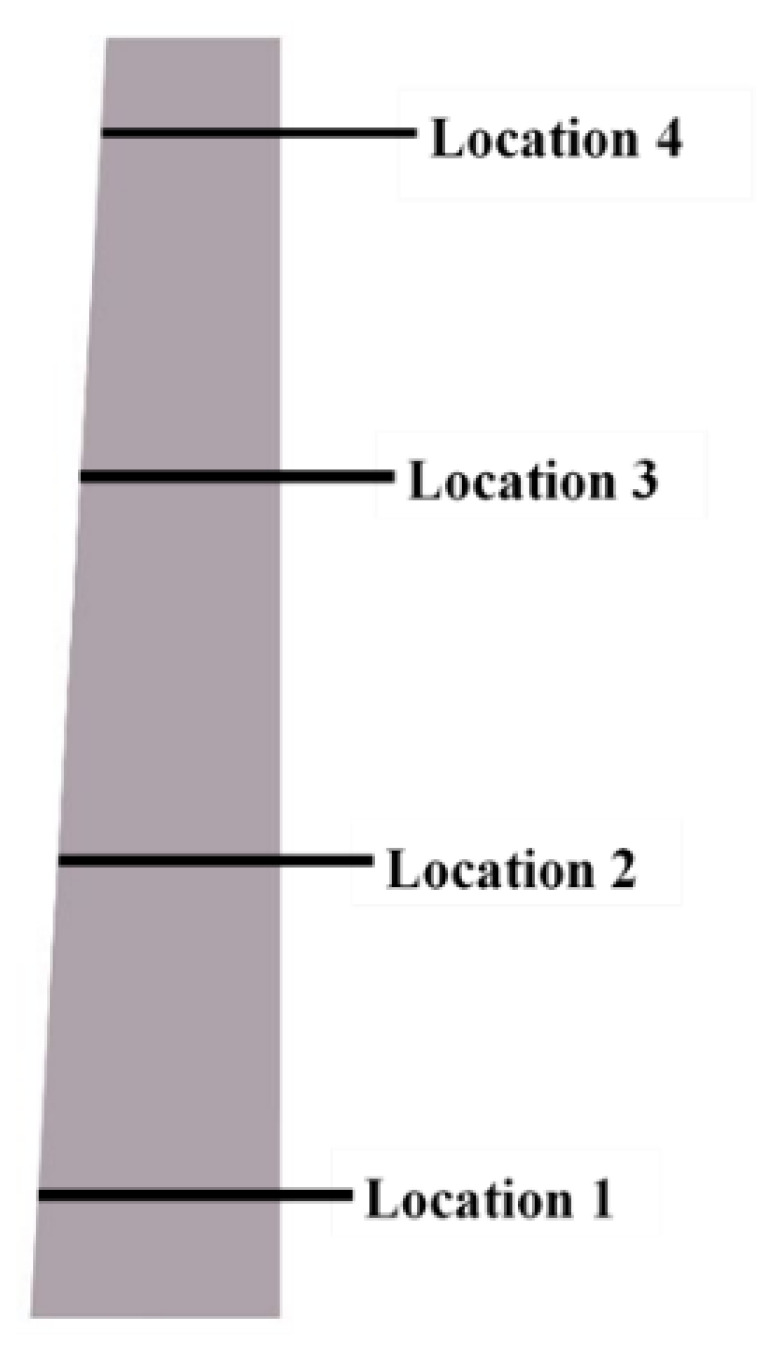
Streamwise locations for radial and tangentially.

Figure 10 shows the streamwise location of diffuser section, which are used to calculate radial and tangential velocity from hub to shroud at each location along the meridional plane for smooth and roughened surfaces. These locations significantly define the behavior of flow at each streamwise location. These streamwise locations are based on flow recirculation and flow reversal positions along the diffuser shroud to hub normalized from 0 to 1. The streamwise location behavior is studied for stall point of the speed line for baseline line compressor and each roughness magnitude.

**Figure 11:**
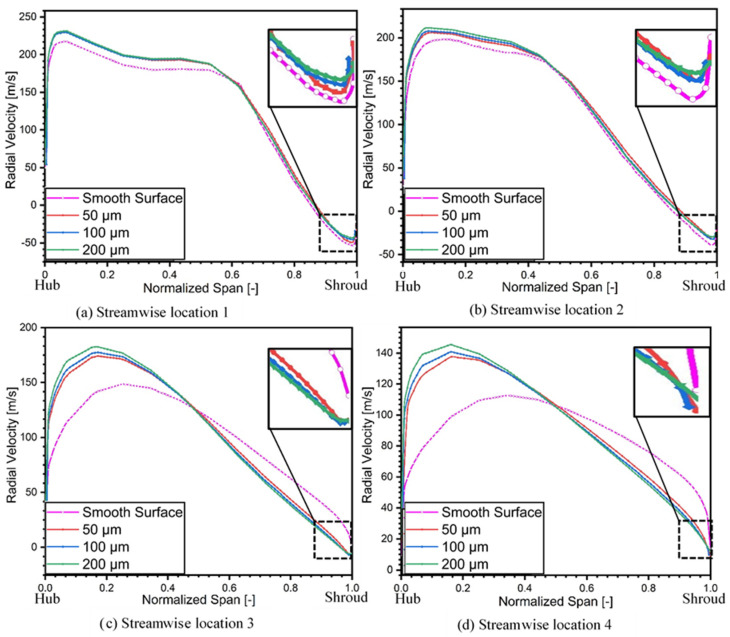
Radial velocity in diffuser section at 4 streamwise locations. (**a**) Location 1. (**b**) Location 2. (**c**) Location 3. (**d**). Location 4.

Figure 11 depicts the influence of different roughness magnitudes on radial spanwise distribution at the meridional plane of diffuser.

As shown in Figure 6, that reverse flow/separated flow zones for all the simulated cases appears at the inlet of diffuser. However, an augmentation in the roughness magnitude reduces the flow reversals. The streamwise location 1 shows maximum flow recirculation near shroud in the smooth surface case as shown by rectangular block in Figure 11a. When roughness has been increased from smooth surface to 200 μm, then it is clear from the magnified view that recirculation has reduced significantly as depicted in Figure 11d, and this phenomenon happens for all the streamwise locations ahead. Thus, increase in the roughness magnitude can reduce the flow separation and flow reversal, which leads to the stabilization of the flow at diffuser shroud.

Even though the case of 50 μm also reduces the flow separation, still there exists a section of small flow separation at the end of the diffuser exit. Furthermore, the maximum radial velocity also moves towards the diffuser hub for all streamwise locations. This transfer in the radial velocity peak is because of the growth in the boundary layer thickness at the shroud surface because of the application of roughness on the diffuser shroud.

It could be seen from the Figure 6 that the flow separation at the outlet of the diffuser exists in the case of the smooth surface while at 200 μm roughness height the flow separation is negligible. The most stable roughness magnitude is 200 μm, however, it significantly effects the performance of the compressor by reducing pressure ratio and efficiency as shown in Figure 9.

**Figure 12:**
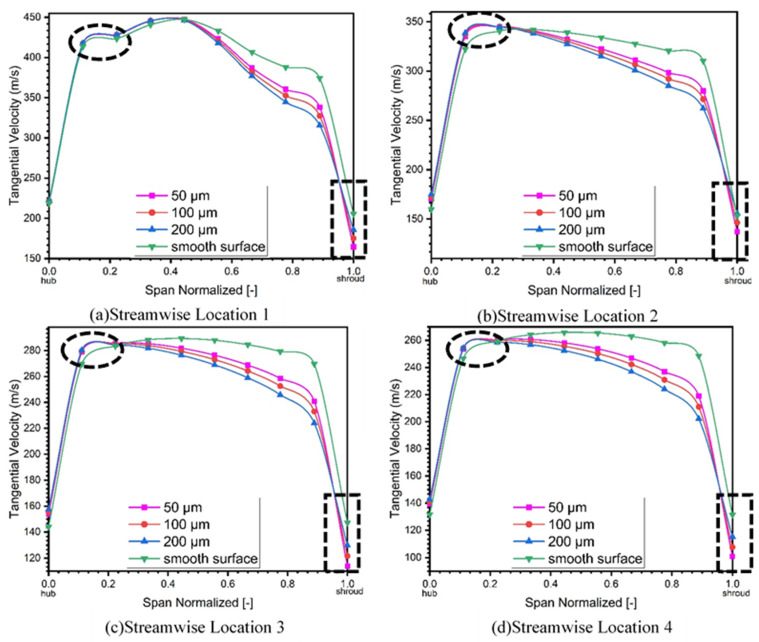
Tangential velocity in diffuser section at 4 streamwise locations. (**a**) Location 1. (**b**) Location 2. (**c**) Location 3. (**d**) Location 4.

Tangential velocity at different streamwise locations along the diffuser’s meridional plane is presented in Figure 12 and has been distributed spanwise at four different locations.

It is evident from the elliptical and rectangular shapes in Figure 12 that increases in surface roughness greatly affect the tangential velocity of the compressor. It could be observed from the rectangle to the extreme right of the graph, that on each streamwise location there is great reduction in the tangential velocity of the compressor with the increase in the roughness magnitude, which results in the overall performance reduction of the turbomachine (i.e., centrifugal compressor) as presented in Figure 12a–d. It is evident from Figure 12a, that tangential velocity drop is much smaller as compared to tangential velocity drop in Figure 12d. By quantification this drop in tangential velocity showed that in Figure 12a, the inlet of diffuser, the drop in tangential velocity for surface roughness of 50 μm is 10%, 15% for 100 μm, and 20% for 200 μm. The drop in tangential velocity for Figure 12d is 14.6% for 50 μm, 21% for 100 μm, and 23% for 200 μm. It means that frictional losses are more significant in case of Figure 12d as compared to Figure 12a. It is concluded from the statistics mentioned above that greater the surface roughness, the higher the drop will be in tangential velocity, and higher the frictional losses will be.

This reduction in the tangential velocity is due to increases in the frictional losses, which counterweighs for the reduction in the performance. It is also evident from the elliptical shape that peak tangential velocity shifts towards the hub for all streamwise locations with increase in surface roughness due to the increase in the boundary layer thickness.

### 4.3. Entropy Generation

Entropy generation is one of the most important factors to show the destruction of useful work in the turbomachinery applications. The efficiency of the compressor is inversely proportional to the entropy generation in the system. The characteristic equation for static entropy generation in the compressor is given below:

(2) $$∆s=c_{p}\ln\left(\frac{T}{T_{\mathrm{ref}}}\right)-\mathrm{Rln}\left(\frac{p}{p_{\mathrm{ref}}}\right)$$

**Figure 13:**
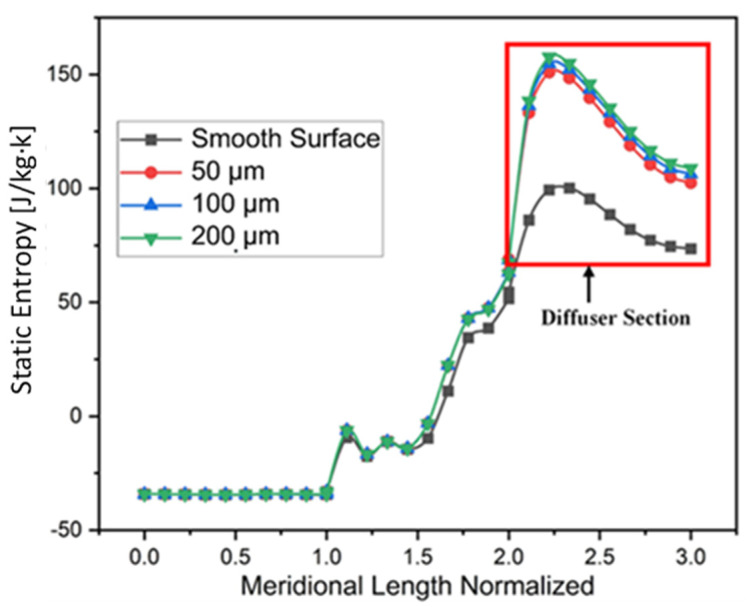
Static Entropy Generation at stall point for different roughness magnitudes.

Figure 13 shows the static entropy generation in the diffuser section of the compressor for all the cases of roughness heights at stall point. It is evident from the figure that as the roughness increase from hydraulically smooth surface to 200 μm, entropy in the diffuser section increases with reduction in the secondary flow which results in the efficiency and pressure loss in the compressor. The impeller flow has relatively very high velocity across the whole compressor due to the entropy generation in the diffuser section being extremely high. In Eckardt’s compressor, one half of the entropy rise occurs in the diffuser section of the compressor [37,38]. Even though there is reduction in secondary flow but with the addition of surface roughness, viscous shear stresses increase near the shroud wall of diffuser, because of which performance of the compressor drops. For 50 μm roughness height, entropy generation rise by 3.5%, 3.7% for 100 μm and 3.9% for 200 μm has been noted.

**Figure 14:**
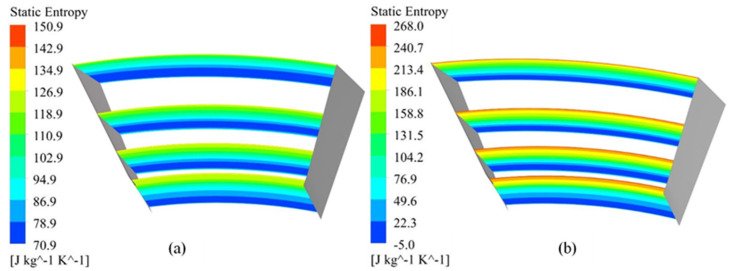
Distribution of static Entropy in diffuser section (**a**) Baseline compressor (**b**) 200 μm roughness magnitude.

To trace the loss mechanism inside the passage of the diffuser, contours for entropy generations are depicted in Figure 14. In case of smooth diffuser shroud shown in Figure 14a, the entropy generation at the shroud is much lower for the baseline compressor, but there is high flow instability due to high flow separation as shown in Figure 6. When the surface roughness increases to 200 μm, it increases the entropy generation at diffuser shroud as shown in Figure 14b, but it helps in flow stabilization and stall control. This increase in the entropy generation at diffuser shroud is attributed to the thick boundary layer due to surface roughness.

In this paper, surface roughness is used as a passive flow control method to control flow instabilities i.e., flow separation and flow recirculation. When roughness magnitude has been increased from 3.18 μm to 200 μm, it is noted that flow recirculation and flow separation has been completely eliminated. Surface roughness is a passive flow control, which implies that it will reduce the performance of the compressor. The pressure ratio and isentropic efficiency has been reduced due to thick boundary layer on diffuser shroud due to surface roughness and frictional losses in diffuser section.

## 5. Conclusions

With the diffuser in focus, parametric analysis of surface roughness on the diffuser shroud and its effect on the performance, operating range and flow structure of the compressor has been studied in this paper using numerical simulations. The major research findings are listed below:

- The compressor has shown peak performance when operated at low mass flow on hydraulically smooth surfaces, which plays important role in the stall margin and operating range of the compressor. At low mass flow rate compressor showed flow instabilities at diffuser inlet.
- To control the flow instabilities, surface roughness has been applied on diffuser shroud to suppress flow separation and hence, stall phenomena.
- The validation results showed overprediction of 2.3% and 8% for SST and k-ε turbulence models, respectively.
- The application of surface roughness increased the operating range from 16% to 18% and stall margin increased from 5.6% to 7.9% by controlling flow separation and flow recirculation effects.
- Based on current analysis, the most efficient design is compressor with surface roughness magnitude of 200 μm, as it completely eliminated the stall phenomena.
- The percentage drop in pressure ratio at stall point for 50 μm is 3.3%, 4.2% for 100 μm and 5.08% for 200 μm using k-ω SST turbulence model.
- The prime contributor to the entropy generation is the relatively high velocity flow in diffuser section with surface roughness applied on the diffuser shroud.
- Entropy generation increased by 3.5%, 3.7% and 3.9% for 50 μm, 100 μm and 200 μm, respectively, due to increased frictional losses and boundary layer thickness.
- Even though, it is clear from the aerodynamics of the compressor that at low mass flow rate, application of surface roughness reduces secondary flow but due to the increased surface roughness, viscous shear stresses deteriorate the performance.
- It is also observed increasing surface roughness increases the percent drop in tangential velocity, and hence increases the frictional losses that leads to the deterioration of the centrifugal compressor performance.

## 6. Future Recommendations

The current steady-state analysis showed effective results as the flow instabilities i.e., flow recirculation and flow separation has been reduced because of the application of surface roughness on diffuser shroud of centrifugal compressor and hence claim further examination. As the flow field in the centrifugal compressor is unsteady, more precise results can be obtained if time-dependent simulations and experimental analysis are performed. It is also recommended to apply other passive flow control methods to reduce flow instability such as vortex generators and creating cavity on diffuser shroud for steady and unsteady simulations.

## Figures and Tables

**Table 1:** Specifications of Baseline Centrifugal Compressor.

Parameter	Value	Unit
Inlet pressure, Pt1	101.3	[kPa]
Inlet temperature, Tt1	15	[°C]
Rotational speed, n	50,000	[rpm]
Mass flowrate at design point, m	2550	[g/s]
Blade flow-angle at inlet (β1)	26.5	degree
Impeller blade tip-speed	586	[m/s]
Impeller pressure-ratio	5.8	-
Isentropic efficiency	84%	-
Full and Splitter blades count	13:13	-
Blade flow-angle at outlet (β2)	52	degree

**Table 2:** Magnitude of Roughness.

Scheme 1.	Roughness Height (μm)
1	50
2	100
3	200

## Data Availability

No new data were created or analyzed in this study. Data sharing is not applicable to this article.
